# Experimental Study of Avalanche Damage Protection Methods for Main Steel Gas Pipelines

**DOI:** 10.3390/ma17133171

**Published:** 2024-06-28

**Authors:** Nurlan Zhangabay, Ulzhan Ibraimova, Timur Tursunkululy, Svetlana Buganova, Arman Moldagaliev, Bolat Duissenbekov

**Affiliations:** 1 Department of Architecture and Urban Planning, M. Auezov South Kazakhstan University, Av. Tauke Khan, No. 5, Shymkent 160012, Kazakhstan; 2 Department of Industrial Civil and Road Construction, M. Auezov South Kazakhstan University, Av. Tauke Khan, No. 5, Shymkent 160012, Kazakhstan; ibraimova_uljan@mail.ru; 3 Department of Building Technologies, Infrastructure and Management International Education Corporation (KazGASA), Ryskulbekov Str., 28, Almaty 050043, Kazakhstan; svetlanabuganova7@gmail.com; 4 Department of Mechanics and Mechanical Engineering, M. Auezov South Kazakhstan University, Av. Tauke Khan, No. 5, Shymkent 160012, Kazakhstan; arm_mold81@mail.ru

**Keywords:** experimental study, avalanche damage, main gas pipeline, steel, winding, crack, temperature loads

## Abstract

This paper conducted an experimental study of reduced models of a main gas pipeline for avalanche damage considering operational conditions. Two options were considered as a method of avalanche damage prevention: single steel rings at the crack edges and steel winding with a winding pitch of 0.25 m. For the tension force, 5% of the steel wire breaking force was taken, which was equal to 1 mm. The ambient environment was simulated by a climatic chamber, where two options of temperature loads were considered: +20 °C and −10 °C. It was found that reinforcement with single rings of pipeline models under conditions of positive (+20 °C) and negative (−10 °C) temperatures showed that the crack opening width in the ring direction decreased 1.63 times and 1.9 times, accordingly. The crack length (longitudinal direction) decreased 2.18 times and 2.45 times, accordingly. The reinforcement of the pipeline models with prestressed wire winding on the crack propagation under conditions of positive (+20 °C) and negative (−10 °C) temperatures showed that the width of the crack opening in the ring direction decreased 1.5 times and 1.46 times, accordingly. The crack length (longitudinal direction) decreased 1.4 times and 1.44 times accordingly, which is a positive moment in addressing the issue of the localisation and stoppage of a crack fracture in main gas pipelines. Simultaneously, the analysis of the prestressed pipelines model test results on crack fracture propagation showed that single rings are more effective, which decreased the crack opening width by 1.1 times and the crack length up to 1.5. Therefore, the experimental results obtained positively complement the available methods of crack localisation in main gas pipelines, which can be used by engineers and research communities when designing or reinforcing existing operating main steel gas pipelines.

## 1. Introduction

**Figure 1:**
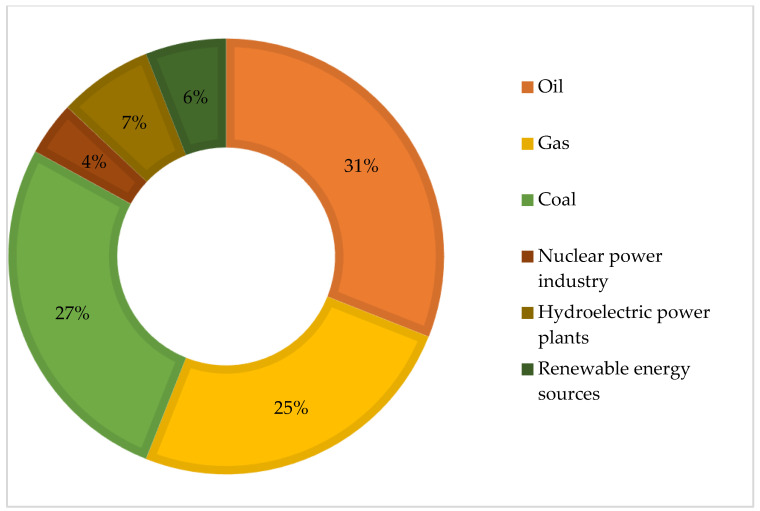
The structure of global energy consumption [1].

Nowadays, gas, like oil, is the main source of energy for many consumers in the world. In recent years, the role and importance of natural gas in the energy balance of the world economy has steadily increased due to its high efficiency as an energy resource and raw material for industry, as well as its higher environmental friendliness compared to oil and coal. Natural gas ranks third after oil and coal in the structure of global energy consumption and, as shown in Figure 1, accounts for 25 per cent of total consumption. 

**Figure 2:**
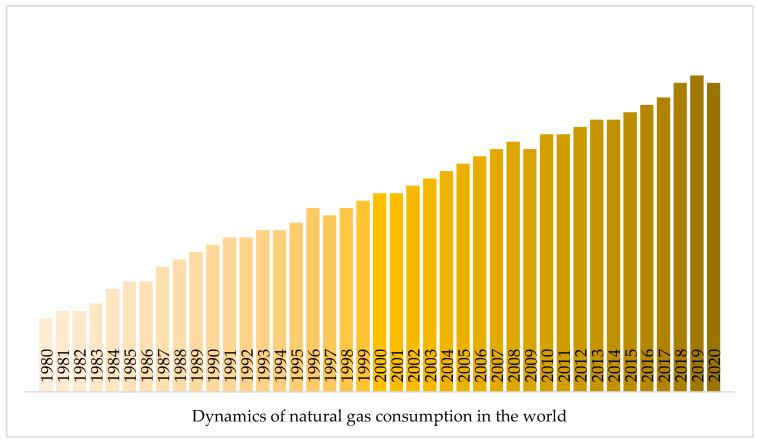
Growth in natural gas consumption from 1980 to 2020 [1].

Over the last 40 years, as shown in Figure 2, the increase in natural gas consumption reached 168%, while the increase in oil consumption over the same period was 44% and coal consumption was 102%. The average growth in gas consumption is 7 per cent per year. 

**Figure 3:**
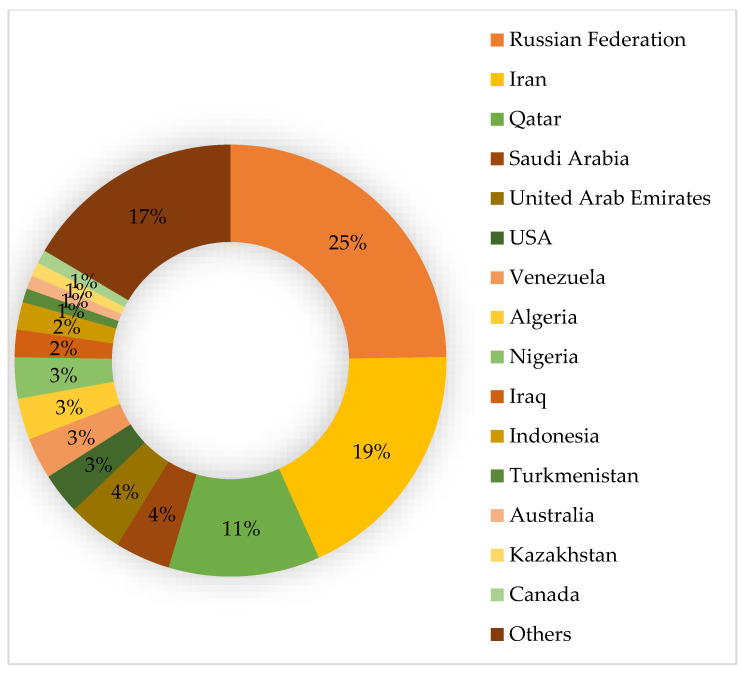
Global natural gas reserves [1].

At present, the world has 138 trillion m^3^ of proven natural gas reserves. The largest share of gas reserves is in Russia (24%), in second place is Iran (18%) and Qatar (11% of the world’s gas reserves) rounds out the top three. Figure 3 shows that Kazakhstan ranks 14th in terms of proven natural gas reserves (2.7 trillion m^3^).

It is logical that global gas exports increased by 78% in the last 20 years, from 528 to 940 billion m^3^. Meanwhile, the share of pipeline gas was 52% or 488 billion m^3^ [1].

Kazakhstan possesses large reserves of natural gas, oil and gas condensate and an extensive network of pipeline transport and has a serious impact on the world energy market development. In this connection, it is necessary to establish a dynamically developing, sustainably functioning and balanced system of pipeline transport in the country as a prerequisite for economic stability and recovery, to ensure the country’s integrity and to improve the population’s living standards. The role of pipeline transport is even stronger under conditions of globalisation of the world economy, which leads to a significant increase in interstate economic relations.

The above data analysis substantiates the obviousness of an intensive construction of steel main gas pipelines, increasing the carrying capacity, increasing the gas pipeline output by the construction of large diameter pipelines and increasing the operating pressures in the pipeline. Their reconstruction and paying great attention to their maintenance in a serviceable and technically suitable condition is a necessity.

Accidents and contingency analyses on main gas pipelines and factors causing their destruction in domestic and foreign sources showed that accidents of different nature occur in main gas pipelines for various reasons [2], where most of the initiators of the failure of main gas pipelines are corrosion of the pipe material, structural defects and the stress concentration in them (seam welds, etc.), as well as external impacts, any of which can lead to avalanche damage of main gas pipelines that can lead to the death of people and environmental damage [3,4,5,6]. 

The problems of pipeline breakage show that intensive research of the issue of oil and main gas pipelines breakage was carried out from the middle of the XX century, resulting in the adopted design standards [7,8,9,10,11,12,13,14,15], but adopted standards mainly regulate the selection of materials, design of units and structures and strength and stability calculations. The strength calculation methods and requirements for pipelines are described in detail. The documents reviewed do not include criteria and methods for assessing the resistance to the occurrence and generation of fracture cracks. No requirements for the assessment of extended fractures of trunk pipelines considering operational loads and impacts have been established. No requirements for design solutions that contribute to effective prevention of avalanche damages in operating and designed pipelines are described.

The first works dedicated to studies of cracking issues in pipelines and methods of their stoppage are dated back to the 1970s [16]. Natural fracture studies of large-diameter pipes were carried out as early as in the 1980s, where the focus was on the methods of pipe manufacture and their mechanical characteristics [17,18]. Since the 1990s, the fracture mechanics theory has been developed and the main crack motion problem, which is initiated by gas motion, has been solved with its involvement [19,20]. In [21,22], the expanded finite element method is used for modelling the stress state near the crack, which has been demonstrated to be accurate and reliable in estimation. 

At the beginning of the XXI century, the modelling of pipeline damage began to involve intensive numerical methods with software systems. The authors of work [23] carried out a number of studies on the application of the finite element method for modelling extended pipeline fractures. The calculation results were used to determine the deformation criterion of fracture mechanics—the crack top opening angle. It was shown that at a critical value of the crack opening, there is a rapid growth of the crack. The literature notes the works that are devoted to methods of localisation and stopping the fracture crack. The article [24] discusses methods of stopping the crack development. It discusses the use of pre-tensioned steel wire winding and composite filaments to localise and stop the fracture crack movement. The article [25] describes a numerical procedure for modelling the gas dynamic processes that are observed during gas flow from a pipe. The works [26,27] present the results of experimental and numerical studies of a gas pipeline section having a bend and working under internal pressure. The study result showed that the critical point under repeated loading is the bending zone, in which a significant stress concentration occurs and consequently a fatigue failure of the pipeline. 

The works on the mathematical description of processes at the propagation of fracture cracks, modelling of various problems of crack propagation dynamics and use of computational complexes and programs have prevailed over the last ten years in the studies of crack formation, propagation and localisation in large-diameter main pipelines. So, in [28], a computational approach to the calculation of the main crack movement in a shell is proposed. The developed programme allows us to consider the dependence of crack propagation on the strain rate in the analysis of pipeline structure failure. The programme also includes plastic deformation of the material at the crack tip and crack development depending on this deformation. It also notes that plastic deformation in a crack top is also initiated by the gas flow action on the banks of a fast-developing crack. 

The article [29] reviews several existing analytical methods of pipeline fracture calculation. The analytical calculation results are compared with the results of an experimental study of a pipeline fracture. It is illustrated that the results obtained will contribute to the development of reliability-based management of pipeline structural integrity. The article [30] gives a review of analytical methods for calculating the fracture of gas pipelines under the action of internal gas pressure in the pipeline. The methods to stop main crack development in gas pipelines are proposed. In work [31], an experience of the European research group on gas pipeline strength is given. It describes the existing approaches to assessing the resistance to the plastic fracture of pipelines. A plastic fracture model is developed based on a numerical analysis of the stress state in the fracture crack zone. The results of a finite element calculation of crack development in a pipeline are considered in [32] and compared with the experimental results of shell models and their failure from rapid crack development. It is demonstrated that the crack propagation rate also depends on the gas decompression rate in the pipeline. A conclusion is made that under certain conditions, the crack propagation rate may be less than the gas decompression rate. Thus, it is pointed out that the gas pipe can stop the propagation of the fracture crack itself. 

The extended finite element method-based study results for gas pipelines of different grades considering operational conditions are given in [33]. In order to examine the initiation and propagation of fracture cracks, defects in the form of corrosion with a depth of 0.5 of the wall thickness were imitated. It was observed that for shorter cracks, the fracture pressure decreased with an increasing initial crack depth. A full-scale experimental study of crack propagation has been studied in works [34,35]. It showed that the dynamic gas pressure at the edge of a propagating crack is lower than the initial pressure up to 7 times. The propagating crack velocity on a long path is up to 2 times lower than the initial velocity. A critical condition of a long fracture is the crack transition from the damage source to a stationary path (at the first metres of the path). Further crack propagation before stopping is a random property. A method of stopping cracks in gas pipelines by changing the configuration (flattening of a gas pipeline wall) of the pipe cross-section when a crack approaches is proposed in the work [36]. 

While describing the fracture mechanism of a gas pipeline, three stages are differentiated: (1) the origin of the crack and its stable state; (2) the development of the crack up to critical dimensions; and (3) the propagation of a quickly developing crack [37]. Prolonged avalanche damage is the final stage of the main gas pipeline fracture process. Gas pipeline cracks can have different shapes and depend on the type of material, internal and external load, causes of occurrence and other factors [2]. Basic classical forms of cracks in main gas pipelines include straight cracks, branching cracks, cross cracks, radial cracks, collapsing cracks, fatigue cracks and corrosion cracks [38]. Straight cracks have a straight shape and can spread along the pipeline without deviation. They are characterised by identical and parallel walls. Straight cracks are the most dangerous from the point of view of extended avalanche damage of main gas pipelines.

Another factor that has a significant role in the process of main gas pipeline failure is the ambient temperature. The reasons for this are thermal stresses in the pipe structure and changes in the mechanical properties of the pipe material at different temperatures (usually at low temperatures). Temperature changes lead to the expansion and contraction of the pipeline materials. It promotes the emergence of additional thermal stresses in the pipe material and becomes dangerous in the presence of defects and damages [39,40]. Such material properties as strength, elasticity and ductility change as the medium temperature changes [41]. This in turn affects the ability of the pipeline to resist mechanical loads and deformations, changing the nature of fracture processes. It is important to understand the influence of temperature on structural strength while developing measures to ensure the safe and reliable operation of gas pipelines with crack-like defects.

The results of an experimental study of fracture crack development in pipelines are of great interest. Thus, the results of experimental studies of fatigue crack development are described in the work [42], and the approaches to accounting for the influence of residual stresses by analytical methods are considered in the review [43].

On the basis of this review, one can conclude that the task of modelling extended avalanche damage in large-diameter gas pipelines considering the factors causing rapid (avalanche) crack propagation is relevant. As of today, the issue of crack propagation along pipeline sections under various operating conditions (internal pressure in the pipeline, medium temperature, design features and soil pattern) is poorly studied and requires further integrated research. 

This conducted review has shown that there are two principal approaches to resolving the problem of preventing extended fractures of gas pipelines. The first is associated with the exclusion of energetic conditions for maintaining the process of stationary crack movement along the pipeline by selecting the parameters of the pipeline material. The second one is associated with using various technological solutions (e.g., the selection of the depth and nature of backfill, changing the mode of operation and operation of a gas pipeline), as well as special structural elements—dampeners and fracture limiters. However, the pipeline operation experience shows that there are situations for which no effective methods of localisation of extended fractures were found, which laid the foundation for the intensive development of constructive and technological methods of prevention of extended gas pipeline fractures.

## 2. Materials and Methods

### 2.1. Setting the Objective and Goals of the Experimental Studies

The scientific and technical literature nowadays contains quite a lot of materials concerning the peculiarities of fractures, crack development and fracture propagation mechanisms, in which different opinions on the mechanism of the gas pipeline fracture and crack propagation rate with regard to temperature effects have been developed. However, due to the difficulties of carrying out tests on the destruction of models and full-scale structures, the transience of the destruction process and parameters measured in the course of an experiment and the necessity of using special methods and experiment techniques and expensive equipment at present, the experimental data on the process of gas pipeline avalanche damage are insufficient.

Based on the information mentioned above, the experiments aimed to reveal the peculiarities of avalanche damages of main gas pipelines of traditional design on reduced models at the first stage and to study and substantiate the possibility of the localisation and stopping of the fracture crack by prestressed winding considering the winding design parameters and operating conditions at the second stage of experiments. 

Regarding this connection, the following tasks were set for the experimental studies:

- To investigate the fracture peculiarities of the linear part models of the traditional main steel gas pipeline under operational conditions, paying attention to the stress state of the fracture zone and the pattern of crack development;
- To investigate the peculiarities of the fracture of the linear part models of the prestressed main steel gas pipeline under operational conditions, paying attention to the stress state of the fracture zone and the pattern of crack development with regard to the constructive parameters of the winding;
- To justify the possibility of fracture crack localisation and stopping by prestressed winding and to assess the influence of the design parameters of prestressing on the fracture crack propagation on reduced models of gas pipelines considering the operational conditions.

In line with the overall objectives of this work, the mechanism of fracture crack propagation will be qualitatively studied at this stage and the applicability of the prestressing method to prevent avalanche damages of gas pipelines will be justified [44,45]. 

### 2.2. Modelling the Geometrical Dimensions of a Gas Pipeline Model

The study of structure operation under different influences with the involvement of different modelling methods has spread to different areas of modern engineering and constitutes a separate field of science, where the main difficulties in modelling shell structures in the form of gas pipelines are related to the thin-wall factor, which significantly complicates the manufacture and testing of models. Therefore, when modelling thin-walled structures under operational conditions, it is attempted to apply models where the wall thickness scale of shell structures is chosen regardless of its overall dimension scale.

This method of modelling is feasible with affine (multi-scale modelling) matching between the pipeline model and the actual structure [46,47]. The affine modelling enables to compose the necessary similarity criteria for thin-walled cylindrical shells at independent linear scales. In this connection, the affine matching between the model and the natural gas pipeline structure was applied to select the geometrical dimensions of a gas pipeline model and their production [48].

This work considers the model fracture with regard to the pattern of operational load application, the model shape and geometrical dimensions, the formed crack size, the material mechanical characteristics of a model and the time. A list of basic parameters is selected in the form of:

(1) $$\underline{l_{1},\;\sigma ,\;\varepsilon ,\;p,\;k,\;\gamma ,\;t,\;E,\;\rho ,\;l,\;\mu }$$

where *l*_1_—crack length, σ—tension, *ε*—relative strain, *p*—internal overpressure, *k*—material viscosity, *γ*—energy released during crack opening, *t*—time, *E*—modulus of elasticity, *ρ*—material density, *l*—characteristic linear dimension and *μ*—Poisson’s ratio.

The dimensionality matrix of the main parameters (1) in the SI system, when excluding dimensionless quantities *ε* and *μ* for the units of force *F*(N), linear dimensions *L*(m) and time *T*(s), will be represented as follows:

(2) $$\begin{array}{c} \begin{array}{cccccccccc} & \;a_{1}\; & \;a_{2}\; & \;a_{3}\; & a_{4} & a_{5} & a_{6} & a_{7} & \;a_{8}\; & \;a_{9}\;\\ & l_{1} & \sigma & p & k & \gamma & \tau & E & \rho & l \end{array}\\ \begin{array}{c} F\\ L\\ T \end{array}\left\|\begin{array}{ccccccccc} 0 & 1 & 1 & 1 & 1 & 0 & 1 & 1 & 0\\ 1 & 2 & -2 & -2 & -1 & 0 & -2 & -4 & 1\\ 0 & 0 & 0 & 1 & 0 & 1 & 0 & 2 & 0 \end{array}\right\| \end{array}$$

Based on the P (Π)-theorem, six independent dimensionless complexes—the similarity criteria—can be formed from 9 basic parameters (2). 

Every dimensionless complex, composed of the basic parameters, is determined from the following formula of dimensions.

(3) $$dim\Pi ={\left(L^{1}\right)}^{a_{1}}{\left(F^{1}L^{-2}\right)}^{a_{2}}{\left(F^{1}L^{-2}\right)}^{a_{3}}{\left(F^{1}L^{-2}T^{1}\right)}^{a_{4}}\;{\left(F^{1}L^{-1}\right)}^{a_{5}}{\left(T^{1}\right)}^{a_{6}}{\left(F^{1}L^{-2}\right)}^{a_{7}}{\left(F^{1}L^{-4}T^{2}\right)}^{a_{8}}{\left(L^{1}\right)}^{a_{9}}$$

For indicators of degree $a_{i}$, there is the following system of algebraic equations:

(4) $$a_{2}+a_{3}+a_{4}+a_{5}+a_{7}+a_{8}=0;\;a_{1}-2a_{2}-2a_{3}-2a_{4}-a_{5}-2a_{7}-4a_{8}+a_{9}=0;\;a_{4}+a_{6}+2a_{8}=0$$

As the result of applying the procedures, we will obtain the following decision matrix for the degree indicators $a_{i}$:

(5) $$\begin{array}{c} \begin{array}{cccccccccc} & \;l_{1}\; & \;\sigma \; & \;p\; & \;k\; & \;\gamma \; & \;t\; & \;E\; & \;\rho \; & \;l \end{array}\\ \begin{array}{c} \Pi_{1}\\ \Pi_{2}\\ \Pi_{3}\\ \Pi_{4}\\ \Pi_{5}\\ \Pi_{6} \end{array}\left\|\begin{array}{ccccccccc} 1 & 0 & 0 & 0 & 0 & 0 & 0 & 0 & -1\\ 0 & 1 & 0 & 0 & 0 & 0 & 0 & 0 & 0\\ 0 & 0 & \;1 & \;0 & 0 & 0 & -1 & 0 & 0\\ 0 & 0 & 0 & 1 & 0 & 0 & -\frac{1}{2} & -\frac{1}{2} & -1\\ 0 & 0 & 0 & 0 & 1 & 0 & -1 & 0 & -1\\ 0 & 0 & 0 & 0 & 0 & 1 & \frac{1}{2} & -\frac{1}{2} & -1 \end{array}\right\| \end{array}$$

By using a matrix (5), the dimensionless complexes of the main parameters are represented in the following form:

(6) $$\Pi_{1}=\frac{l_{1}}{l};\;\Pi_{2}=\frac{\sigma }{E};\;\Pi_{3}=\frac{p}{E};\Pi_{4}=\frac{k^{2}}{E\rho l^{2}};\;\Pi_{5}=\frac{\gamma }{El};\;\Pi_{6}=\frac{t^{2}E}{\rho l^{2}.}$$

In order to obtain a complete system of dimensionless complexes, we include the Poisson’s ratio μ and relative strain ε in the list (6) and write the following conditions of invariance of the similarity criteria as the required one:

(7) $$\Pi_{1}=\frac{l_{1}}{l}=idem;\;\Pi_{2}=\frac{\sigma }{E}=idem;\;\Pi_{3}=\frac{p}{E}=idem;\;\Pi_{4}=\frac{k^{2}}{E\rho l^{2}}=idem;\;\Pi_{5}=\frac{\gamma }{El}=idem;\;\Pi_{6}=\frac{t^{2}E}{\rho l^{2}}=idem;\;\Pi_{7}=\varepsilon =idem;\;E_{8}=\mu =idem.$$

where *idem* means that the relevant dimensionless relation for a given process should remain unchanged. 

It is useful to write condition (7) in the following modified form:

(8) $$\begin{array}{l} \Pi_{1}^{*}=\Pi_{1}=\frac{l_{1}}{l};\;\Pi_{2}^{*}=\Pi_{2}=\frac{\sigma }{E};\;\Pi_{3}^{*}=\Pi_{3}=\frac{p}{E};\\ \Pi_{4}^{*}=\Pi_{4}=\frac{k^{2}}{E\rho l^{2}};\;\Pi_{5}^{*}=\Pi_{5}=\frac{\gamma }{El};\;\Pi_{6}^{*}=\Pi_{6}=\frac{tE}{\rho l^{2}};\\ \Pi_{7}^{*}=\Pi_{7}=\varepsilon ;\;E_{8}^{*}=E_{8}=\mu \end{array}$$

By using (8), the dependences between the crack length *l*_1_, stresses *σ*, strains ε and the process-determining parameters are written in the following criterion form:

(9) $$\frac{l_{1}}{l}=f_{1}\left(\frac{P}{E},\frac{t^{2}E}{\rho l^{2}},\frac{k^{2}}{E\rho l^{2}},\frac{\gamma }{El},\mu \right);\;\frac{\sigma }{E}=f_{2}\left(\frac{p}{E},\frac{t^{2}E}{\rho l^{2}},\frac{k^{2}}{E\rho l^{2}},\frac{\gamma }{El},\mu \right);\;{\varepsilon =f}_{3}\left(\frac{p}{E},\frac{t^{2}E}{\rho l^{2}},\frac{k^{2}}{E\rho l^{2}},\frac{\gamma }{El},\mu \right).$$

The conditions (9) in accordance with the definition of the similarity of two phenomena occurring in the model (*m*) and the real structure (*t*) are written in the form:

(10) $$\frac{l_{1,m}}{l_{m}}=\frac{l_{1,t}}{l_{t}};\;\frac{\sigma_{m}}{E_{m}}=\frac{\sigma_{t}}{E_{t}};\;\frac{p_{m}}{E_{m}}=\frac{p_{t}}{E_{t}};\varepsilon_{m}=\varepsilon_{t};\;\mu_{t}=\mu_{m};\;\;\frac{{k^{2}}_{t}}{E_{t}\rho_{t}{l^{2}}_{t}}=\frac{{k^{2}}_{m}}{E_{m}\rho_{m}{l^{2}}_{m}};\frac{\gamma_{t}}{E_{t}l_{t}}=\frac{\gamma_{m}}{E_{m}l_{m}};\;\frac{t_{m}^{2}E_{m}}{\rho_{m}l_{m}}=\frac{t_{t}^{2}E_{t}}{\rho_{t}l_{t}};$$

If we assume that the model of material corresponds to the material of a real structure, then for the elastic modulus *E*, Poisson’s ratio μ and material density *ρ*, we can take

(11) $$\frac{E_{t}}{E_{m}}=1;\;\frac{\rho_{t}}{\rho_{m}}=1;\;\frac{\mu_{t}}{\mu_{m}}=1$$

Given (11) and assuming the modelling scale of linear dimensions $m_{l}=l_{t}/l_{m},$ the expression (10) in the final form, through the similarity coefficients, will be written in the following form: 

For the crack length, $m_{l1}=\frac{l_{1m}}{l_{1t}}=\frac{l_{m}}{l_{t}}=\frac{1}{m_{l}}$;

For the material viscosity, $m_{k}=\frac{k_{m}^{2}}{k_{t}^{2}}=\frac{l_{m}^{2}}{l_{t}^{2}}=\frac{1}{m_{д}^{2}}$;

For the tensions, $m_{\sigma }=\frac{\sigma_{m}}{\sigma_{t}}=1$;

For the energy released during the crack opening, $m_{\gamma }=\frac{\lambda_{m}}{\lambda_{t}}=\frac{l_{m}}{l_{t}}=\frac{1}{m_{l}}$;

For the time, $m_{t}=\frac{t_{m}^{2}}{t_{t}^{2}}=\frac{l_{m}^{2}}{l_{t}^{2}}=\frac{1}{m_{l}^{2}}$.

The formulae obtained made it possible to transfer with satisfactory convergence the results of the gas pipeline models’ tests, performed in scale up to 1:5, to the design of a full-scale gas pipeline.

### 2.3. Description of the Main Gas Pipeline Model Designs, Measuring Equipment and Installations

The methodology of experimental research is based on a set of rules, consisting of the reproduction of operational, technological, geometric and design parameters with a maximum recreation of real conditions, with the use of appropriate test equipment, measuring instruments and apparatus, as well as appropriate modern processing software systems. A model of the main steel gas pipeline was chosen as the object of research, which was produced based on the similarity criteria of the model and the natural tank. The main geometric dimensions of the model and the test series of the main steel gas pipeline are shown in Table 1.

A chemical composition of the main gas model material and steel winding was determined by laser analysis using an elemental laser analyser by X-ray fluorescence analysis according to [49], the results of which are shown in Table 2 and Table 3.

As the mechanical properties of the steel material change insignificantly depending on the ambient temperature [39,40], in this experimental study, the material properties at a temperature equal to 20 °C were chosen according to [50,51,52,53,54] (Table 4).

To achieve the experimental research objectives, the following design variants were modelled based on the main gas pipeline model: 1—the traditional construction of the main gas pipeline (without prestressing); 2—the main gas pipeline with a prestressed steel ring at the crack tops; and 3—the main gas pipeline with prestressed wire winding with a winding thread pitch of 0.25 m and an angle of 90°.

**Figure 4:**
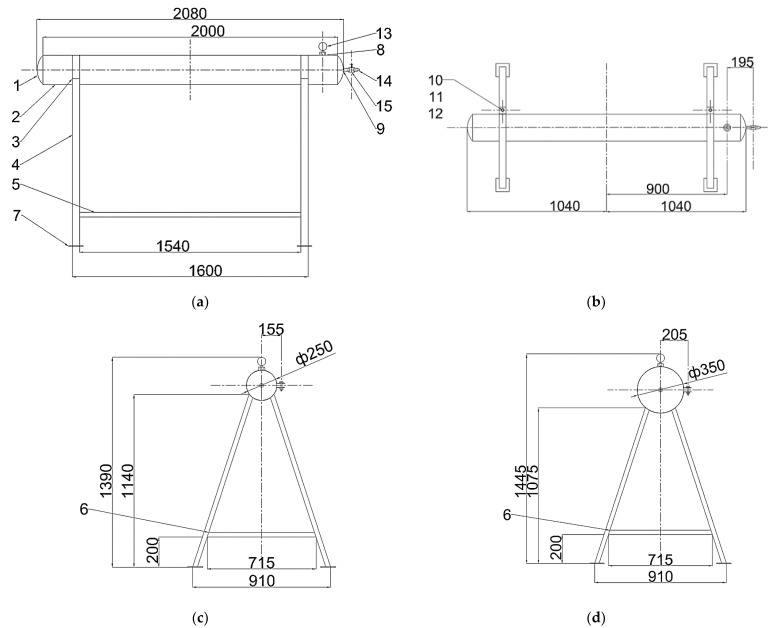
Structural diagrams of main gas pipeline models: (**a**)—side view; (**b**)—top view; (**c**)—front view (pipe diameter 250 mm); and (**d**)—front view (pipe diameter 350 mm).

**Figure 5:**
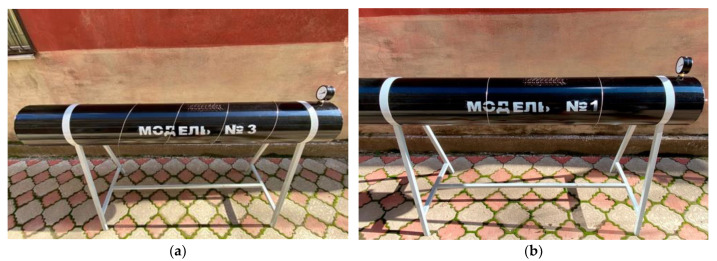
Model of prestressed gas pipeline: (**a**)—tension of steel wire with a certain winding pitch; (**b**)—tension of single steel rings.

The general view of the reduced models of a traditional gas pipeline prepared for testing and their design scheme are presented in accordance with Figure 4 and Table 5. Two models with an internal diameter of 250 mm and 350 mm were considered as the diameter of the reduced gas pipeline. A reduced model of a prestressed gas pipeline is shown in Figure 5.

Reduced models of a prestressed main steel gas pipeline were manufactured in stationary ones, where the tension force of the winding thread was assumed to be 0.05 σU, where σU is the wire breaking force (12), Figure 5. At the same time, this study considered two types of reinforcement methods, that is, the tension of a steel wire with a certain winding pitch of 0.25 m (Figure 5a) and the tension of single steel rings along the edges of the crack (Figure 5b). A steel wire with a diameter of 1 mm was chosen as the winding.

**Figure 6:**
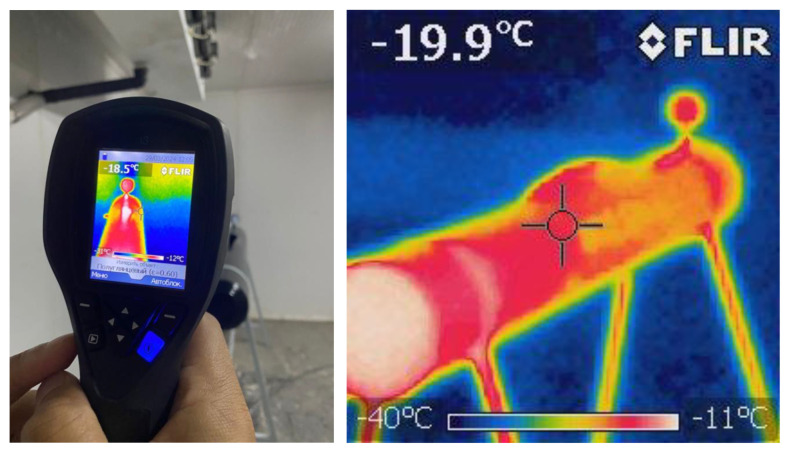
Thermograms of a main steel gas pipeline model captured by FLIR thermal imaging camera in a “Panelsystems” climate chamber.

While investigating the ambient temperature effect on the nature of a pipeline fracture, the pipeline models were placed in the Panelsystems climatic chamber (the dimensions of the chamber are 7 × 5 × 4 m), which is able to simulate the temperature range from +70 °C to −50 °C. Figure 6 shows the thermograms of the pipeline model surface taken by the FLIR thermal imager. The surface temperatures of the pipeline model, +20 °C and −10 °C, were modelled in the experiment. 

The measuring instruments and primary voltage transducers were chosen with regard to the expected values of the measured test parameters, ambient temperature and humidity and based on the recommendations [55,56,57].

As per the experimental study goals, the stress–strain state of the model wall along the banks of the fracture crack was evaluated by testing the models; the dimensions (angle, length, width and opening) of the crack were determined and the nature of the fracture crack development was studied.

Internal pressure was applied by injecting air into the model using a FINIWARRIORBK-113-3M-AP compressor (FPS Compressors, Oxford, UK) and monitored with a 0.1 MPa manometer. A fracture point was created artificially by milling a longitudinal notch 100 mm long and half the wall thickness (0.4 mm) into the model body.

The stress–strain state of the wall in a fracture area was evaluated by strain measurement methods [58]. Strain gauges of the BF350-4BB (11)N6-F-X-V2 brand were used as the primary transducers.

**Figure 7:**
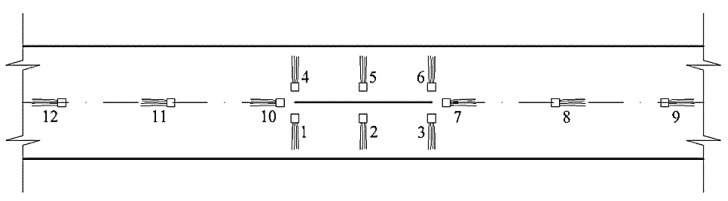
Strain gauge sticking scheme in the fracture crack development zone.

Strain gauges were glued in the crack propagation area, in accordance with Figure 7, so that it was possible to measure the ring and longitudinal strains at the experimental point.

**Figure 8:**
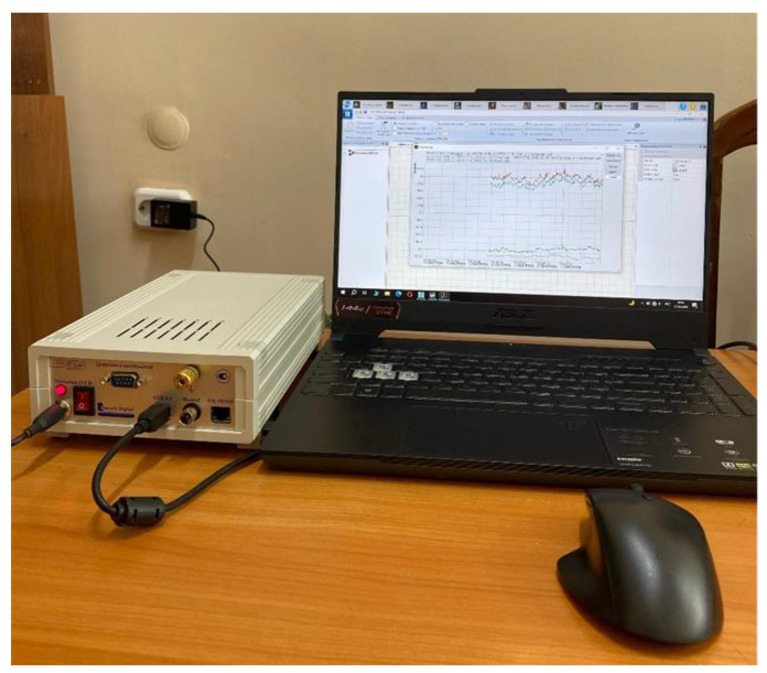
Secondary strain measurement transducers.

A 03-53 strain gauge mounting kit, ZET 7010 multichannel measuring module and ZET 7076 intelligent interface converter were used for the secondary strain measurement transducers (Figure 8).

### 2.4. Methods for Conducting the Experiments to Study Avalanche Damages on Main Gas Pipeline Models

While testing the pipeline models, the following coil parameters were modelled: the pitch, steel coil force and steel coil diameter. The crack dimensions, relative deformation, internal overpressure and temperature at the pipe surface were measured and recorded by the tests.

Prestressing was imitated by winding a high-strength wire of 1.0 mm diameter on the body of a gas pipeline model under steady-state conditions with a tension force equal to 0.05 σ_U_ (of the winding wire breaking force) and a winding step equal to a = 0.25 m. The steel rings were placed at the tops of non-cross cracks with a similar tension force and thread diameter.

The force of the ring tension is determined by the following expression [59]:

(12) $$N=0.25\pi {\;}^{2}{\cdot k\cdot \sigma }_{U}.$$

where k—the coefficient from the steel wire critical breaking force.

**Figure 9:**
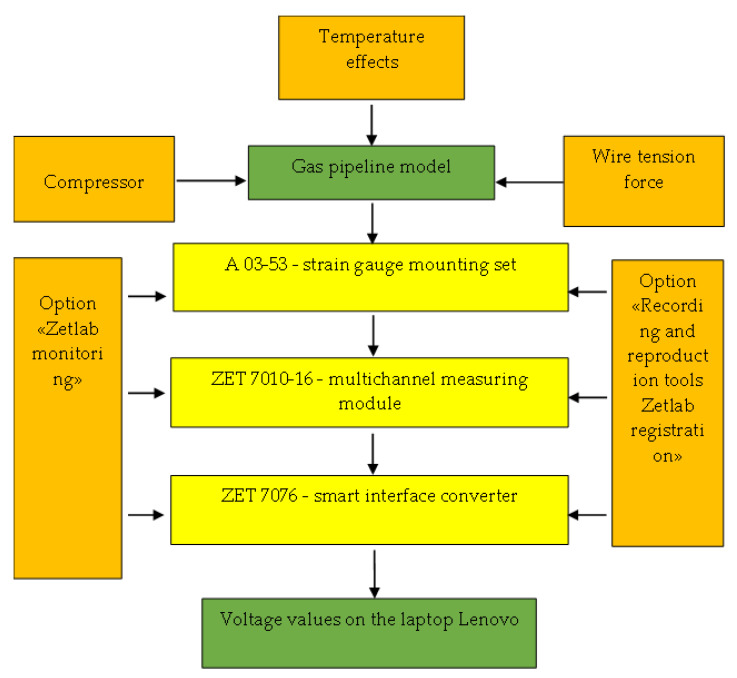
Experimental complex flow chart.

The experimental testing flowchart of the main steel gas pipeline model for avalanche damage with regard to temperature effects is presented in Figure 9.

The crack opening pattern was determined during the testing of the traditional main pipeline models, and the crack opening width and crack length were measured under different operating conditions. To compare the test results of a traditional design model with those of prestressed pipeline models, all the model tests were carried out under similar conditions. The models were tested at temperatures of +20 °C and −10 °C for the assessment of the pipeline surface temperature influence on the crack propagation pattern. The results of the pipeline model tests were used to assess the effectiveness of the prestressing method in main gas pipelines to prevent avalanche damage of the gas pipelines. The experimental data were defined in accordance with the recommendations [60,61].

## 3. Results and Discussion

A total of 12 series of main gas pipeline models were tested in the experiment. The experimental studies were performed in three stages: stage 1—the model tests of the traditional design pipeline (without winding); 2—the model tests of the pipeline prestressed by rings; and 3—the tests of the model of the pipeline prestressed by wire winding. The characteristics of an initial crack in all the models are the same, the geometric dimensions (length/width/depth) of which are equal to 100/1.0/0.4 mm.

**Figure 10:**
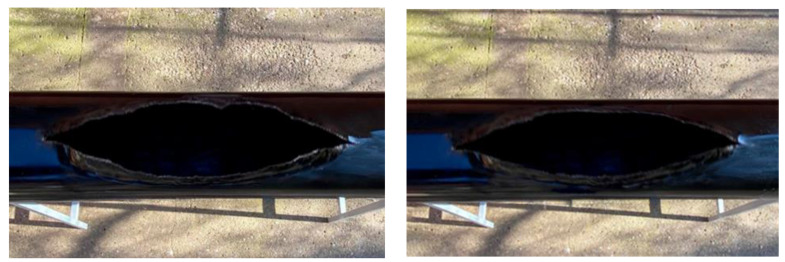
Fracture crack pattern in a traditional pipeline model.

**Figure 11:**
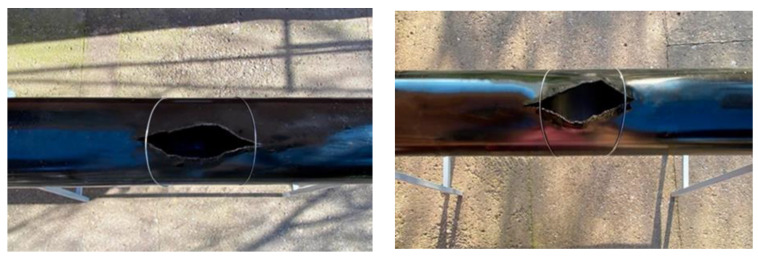
Fracture crack pattern in a prestressed ring pipeline model.

The test results of the traditional gas pipeline model (without winding) as per the test series I-A, II-A, III-A and IV-A are presented in Table 6, Table 7 and Table 8, and the crack opening pattern is presented in Figure 9, Figure 10 and Figure 11.

The experimental result of the traditional gas pipeline model under temperature conditions of +20 °C showed (Figure 10) that in the pipeline model with a diameter of 250 mm (series I-A) at an internal critical pressure of 1.519 MPa, the crack opening width was 183 mm, and the crack length in a longitudinal direction deformation occurs in the form of a crack extension up to 375 mm (Table 6). Similar to the design study, the maximum stresses occur in the crack centre area in the ring direction, and the longitudinal stresses are much smaller than the ring stresses, which is consistent with the cylindrical shell theory of operation under pressure. 

The same pattern of fracture was observed at a negative temperature equal to −10 °C (series II-A). The crack opening width was 165 mm (in the annular direction), and the length (in the longitudinal direction) was 354 mm (Table 6). It showed that the surface temperature of −10 °C does not significantly affect the pattern of crack propagation and its stress state.

Similar crack propagation patterns were observed in the series III-A and IV-A testing of a 350 mm diameter model. The crack opening width (in ring direction) at +20 °C and −10 °C was 235 mm and 226 mm, respectively, and the crack length was 416 mm and 398 mm, respectively (Table 6). 

The fracture crack opening pattern in the prestressed ring test series is shown in Figure 11.

The tests revealed that the crack opening pattern in the test series did not change. But the crack opening in the ring direction of a 250 mm diameter model (series I-B) was 112 mm and 109 mm at the surface temperatures of +20 °C and −10 °C, and the crack length (longitudinal direction) was 172 mm and 144 mm, respectively. A similar propagation pattern for series III-B and IV-B was observed: the crack opening in the ring direction was 151 mm and 141 mm, respectively, and in the longitudinal direction it was 215 mm and 203 mm.

**Figure 12:**
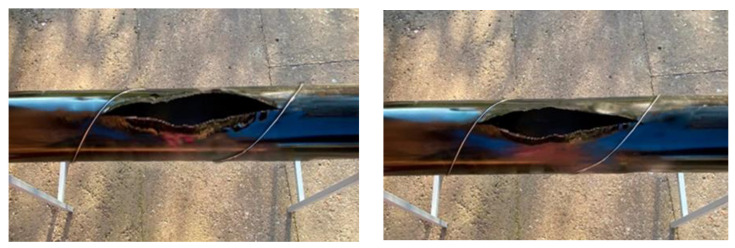
Fracture crack pattern in a prestressed wire wound pipeline model.

The third stage involved the testing of wire-wound pipeline models with a winding filament pitch of 0.25 m for the test series I-B, II-B, III-B and IV-B. Figure 12 illustrates the pattern of the fracture crack propagation in the model of the prestressed wire-wrapped pipeline.

The pattern of the crack opening, as shown in Figure 12, has changed compared to the test results of the pipeline model without wrapping. The crack width in a 250 mm diameter pipeline model reached 121 mm and the length reached up to 264 mm at a surface temperature of +20 °C. At the negative temperature, the crack width was 129 mm and the length reached up to 250 mm (series II-B). Similar results were obtained for the tests on a 350 mm diameter pipeline model. The crack width reached the dimensions of 165 mm and the length reached up to 296 mm (series III-B). 

The analysis of the deformation and crack opening pattern above showed that under operational conditions, using the method of crack localisation in the form of single steel rings at the vertices in a model with a diameter of 250 mm, the crack opening width in the ring direction decreased from 183 mm to 113 mm (1.63 times), and the crack length decreased from 375 mm to 172 mm (2.18) times, which can be assessed as a positive effect. 

Under the conditions of a negative temperature (−10 °C), the crack opening width in the ring direction decreased from 165 mm to 109 (1.51 times), and the crack length decreased from 355 mm to 144 mm (2.45 times). 

Roughly the same reduction was obtained from prestressing with single rings in the case of the 350 mm diameter pipeline tests. The crack opening width decreased 1.5–1.6 times and the crack length 1.9–1.95 times depending on the operating temperature.

The efficiency assessment of the steel winding application for the localisation and stoppage of a fracture crack in main gas pipelines showed that under conditions of a positive temperature, the steel winding localises and stops crack propagation in an annular direction from 183 mm to 121 mm (1.5 times) at the gas pipeline model diameter of 250 mm. The longitudinal crack propagation (length) decreased from 376 mm to 264 mm (1.42 times). When the surface temperature of the model surface is negative (−10 °C), the reduction in the crack opening in the ring direction was from 165 mm to 129 mm (1.27 times), and the reduction in the crack length in the longitudinal direction was from 355 mm to 250 mm, which is 1.41 times less. A similar qualitative effect was observed in the model pipeline with a diameter of 350 mm, where the reduction in the crack width was 1.46 times, and the crack length was 1.44 times. 

At single prestressed rings, the crack opening width decreased by a factor of 1.57 and the crack length in the longitudinal direction by a factor of 2.12. The steel wire winding reduced the crack opening width 1.42 times and the crack length 1.41 times. The test results analysis of the prestressed pipeline models on fracture crack propagation showed that single rings are more effective, which reduced the crack opening width by 1.1 times and the crack length by 1.5 times. 

## 4. Conclusions

1. Based on the mechanical similarity theory and affine modelling, the structures of a natural main pipeline were modelled, and similarity criteria were specified. The similarity coefficients obtained allowed for satisfactorily transferring the experimental results to real gas pipeline structures. A modelling scale up to M1:5 was chosen in accordance with the experiments’ objective, as well as with consideration of specific features in the production and testing of thin-walled structures. The selected experimental methodology, measuring devices and equipment allowed for fully solving the goal set before the experiments—to justify the possibility of using the method of prestressing for the localisation and stopping of fracture cracking.
2. The test results of the prestressed single-ring pipeline models under conditions of positive (+20 °C) and negative (−10 °C) temperatures showed that the crack opening width in the ring direction decreased 1.63 times and 1.9 times, accordingly. The crack length (longitudinal direction) decreased 2.18 times and 2.45 times, accordingly. It is noted that it can be considered as a positive moment in the solution of the localisation and stopping of fracture cracking in main gas pipelines.
3. The experimental study results of the pipeline models prestressed with wire winding on fracture crack propagation under conditions of positive (+20 °C) and negative (−10 °C) temperatures showed that the crack opening width in the ring direction decreased up to 1.5 times and 1.46 times, accordingly. The crack length (longitudinal direction) decreased 1.4 times and 1.44 times, accordingly. It can be considered as a positive moment in the solution of the localisation and stopping of fracture crack in main gas pipelines.
4. The test results analysis of the prestressed pipeline models on the fracture crack propagation showed that single rings are more effective, which reduced the crack opening width by 1.1 times and the crack length to 1.5.
5. The stress–strain state analysis of the crack propagation area showed that the stress buildup starts in the crack’s middle and contributes to the emergence of a flaw and rapid crack propagation. The stress–strain pattern of the crack area shows an increase in ring stresses in the pipeline wall.

## Figures and Tables

**Table 1:** Geometric dimensions of the main steel gas pipeline model and the test series.

No.	Test Series	Model Length, mm	Model Diameter, mm	Model Wall Thickness, mm	Steel Winding Thickness, mm	Type of Prestressing	Wall/Wire Winding Material	Test Temperature, °C	
1	I-A	2000	250	0.8	1	Without winding	St3kp/St2kp	+20 °C	
2	I-Б	2000	250	0.8	1	Steel ring		+20 °C	
3	I-B	2000	250	0.8	1	Wire winding		+20 °C	
4	II-A	2000	250	0.8	1	Without winding			−10 °C
5	II-Б	2000	250	0.8	1	Steel ring			−10 °C
6	II-B	2000	250	0.8	1	Wire winding			−10 °C
7	III-A	2000	350	0.8	1	Without winding		+20 °C	
8	III-Б	2000	350	0.8	1	Steel ring		+20 °C	
9	III-B	2000	350	0.8	1	Wire winding		+20 °C	
10	IV-A	2000	350	0.8	1	Without winding			−10 °C
11	IV-Б	2000	350	0.8	1	Steel ring			−10 °C
12	IV-B	2000	350	0.8	1	Wire winding			−10 °C

**Table 2:** Chemical composition of the tank model structural steel.

Name	Content, %										Steel Grade by Grade Book
	CE	Fe	C	Mn	Mo	Ni	Cr	V	Cu	Al	
Pipe model material	0.281	99.242	0.213	0.305	-	0.073	0.043	0.009	0.026	0.028	St3kp

**Table 3:** Chemical composition of steel wire.

Name	Content, %										Steel Grade by Grade Book
	CE	Fe	C	Mn	Mo	Ni	Cr	V	Cu	Al	
Steel winding material	0.187	99.374	0.122	0.266	-	0.089	0.052	0.009	0.042	0.010	St2kp

**Table 4:** Mechanical characteristics of pipe and winding model material [50,51,52,53,54].

Name of Characteristic	Steel	$\mathrm{Ultimate}\;\mathrm{Strength}\;\sigma_{B}$, MPa	δ	ψ	Hardness HB, No More
			%		
Pipe model material	St3kp	363	23	-	131
Material of steel winding	St2kp	324	25	-	116

**Table 5:** The structural parts of a model and their geometric dimensions.

No.	Name	Dimension	Quantity	Dimension	Quantity
1	Cylindrical lid 0.8 mm	250	2	350	2
2	Pipe 200 × 0.8 m	2000	1	2000	1
3	Clamp 50 × 2	250	2	350	2
4	Pipe 30 × 30 × 2.0 mm	1195	4	1125	4
5	Pipe 30 × 30 × 2.0 mm	1540	1	1540	1
6	Pipe 30 × 30 × 2.0 mm	715	2	715	2
7	Sheet 100 × 100 × 4.0 mm		4		4
8	Manometer pipe	m12 × 1.5	1	m12 × 1.5	1
9	Crane pipe	1/4’	1	1/4’	1
10	Pin M10 × 65		2		2
11	Ring plate M10		4		4
12	Nut M10		2		2
13	Manometer 16kPa		1		1
14	Air compressor connector		1		1
15	Valve 1/4’		1		1

**Table 6:** Results of calibration experimental studies.

No.	Series	Tension Force, kN (0.05 σ_u_)	Winding, without Winding/Ring/Wire	Model Critical Pressure, MPa	Initial Crack Parameters, mm	Crack Width after Rupture, mm	Crack Length after Rupture, mm
1	I-A	-	+/−/−	1.519	100/1.0/0.4	183.3	375.6
2	I-Б	0.13	−/+/−	1.554	100/1.0/0.4	112.5	172.2
3	I-B	0.13	−/−/+	1.598	100/1.0/0.4	120.5	264.1
4	II-A	-	+/−/−	1.553	100/1.0/0.4	165.2	354.8
5	II-Б	0.13	−/+/−	1.584	100/1.0/0.4	108.8	144.5
6	II-B	0.13	−/−/+	1.618	100/1.0/0.4	129.2	250.2
7	III-A	-	+/−/−	1.237	100/1.0/0.4	235.4	416.4
8	III-Б	0.13	−/+/−	1.253	100/1.0/0.4	151.2	215.4
9	III-B	0.13	−/−/+	1.319	100/1.0/0.4	164.5	296.1
10	IV-A	-	+/−/−	1.28	100/1.0/0.4	226.6	398.7
11	IV-Б	0.13	−/+/−	1.291	100/1.0/0.4	141.1	203.5
12	IV-B	0.13	−/−/+	1.275	100/1.0/0.4	154.4	275.5

**Table 7:** Strain gauge values as per test series with respect to Figure 7 with respect to ring stresses.

No.	Series	Stress Value in Ring Direction, MPa					
Strain Gauges		1	2	3	4	5	6
1	I-A	11.534	11.035	10.597	12.021	11.201	10.907
2	I-Б	12.203	12.784	15.114	11.988	12.002	16.021
3	I-B	12.245	12.652	15.442	12.021	12.112	15.887
4	II-A	12.851	12.327	12.015	13.042	12.984	11.883
5	II-Б	12.314	12.941	16.022	12.547	12.215	16.571
6	II-B	12.578	13.095	15.991	12.641	13.874	15.801
7	III-A	10.525	10.358	10.124	10.689	10.401	10.205
8	III-Б	11.541	12.014	13.247	11.674	11.985	13.022
9	III-B	11.749	12.025	14.014	11.877	12.146	13.998
10	IV-A	10.954	10.572	10.012	10.851	10.382	10.985
11	IV-Б	12.041	12.545	13.778	12.005	11.974	13.254
12	IV-B	12.142	12.447	14.002	12.875	12.245	14.113

**Table 8:** Strain gauge values as per test series with respect to Figure 7 with respect to longitudinal stresses.

No.	Series	Stress Value in Ring Direction, MPa					
Strain Gauges		7	8	9	10	11	12
1	I-A	0.110	0.051	0.013	0.063	0.041	0.011
2	I-Б	0.141	0.097	0.011	0.132	0.084	0.009
3	I-B	0.175	0.087	0.013	0.141	0.081	0.005
4	II-A	0.113	0.067	0.019	0.088	0.067	0.013
5	II-Б	0.157	0.101	0.015	0.141	0.091	0.011
6	II-B	0.121	0.105	0.005	0.132	0.099	0.001
7	III-A	0.075	0.061	0.002	0.055	0.031	0.005
8	III-Б	0.081	0.042	0.005	0.073	0.052	0.001
9	III-B	0.071	0.051	0.001	0.069	0.032	0.006
10	IV-A	0.086	0.065	0.004	0.079	0.046	0.008
11	IV-Б	0.088	0.051	0.008	0.079	0.049	0.002
12	IV-B	0.067	0.042	0.002	0.068	0.045	0.006

## Data Availability

The original contributions presented in the study are included in the article, further inquiries can be directed to the corresponding authors.
